# Infusing Representativeness and Cultivating Harmonization in Alzheimer’s Trials: World Wide FINGERS

**DOI:** 10.1093/geroni/igab046.257

**Published:** 2021-12-17

**Authors:** Rema Raman, Neelum Aggarwal

## Abstract

World Wide Fingers is a network involving over 30 countries organized to conduct randomized controlled clinical trials to slow the progression of cognitive decline and reduce dementia risks. Trials are designed to parallel the successful Finnish Geriatric Intervention Study to Prevent Cognitive Impairment and Disability (FINGER) trial of a multidomain lifestyle intervention featuring increased physical activity, improved diet, cognitive training, and metabolic risk factor monitoring. While FINGER found that its intervention significantly benefited cognitive function, it is not clear whether this approach might be successfully tailored to other cultures and environments to yield similar results. This is the goal of World Wide FINGERS. It infuses representativeness by enrolling cohorts that reflect the communities in which it is conducted. For findings across the many trials to be integrated, it is necessary for protocols to be harmonized as much as possible. The COVID-19 pandemic presents special challenges towards harmonization as its disruptions of trial protocols and conduct vary among countries and over time. This symposium is organized to provide the scientific background and framework for the World Wide FINGERS. Novel grassroots efforts towards enrolling representative cohorts in the US will be described. Plans for harmonization and federated data analyses spanning international boundaries and regulations will be outlined. Integrated approaches to challenges of COVID-19 pandemic across trials will be presented. The conclusion of this session will be a discussion of how World Wide FINGERS may serve as a model for collaborative approaches to identify effective, translatable approaches to reduce risks for Alzheimer’s disease.

